# Effect of IMU Design on IMU-Derived Stride Metrics for Running

**DOI:** 10.3390/s19112601

**Published:** 2019-06-07

**Authors:** Michael V Potter, Lauro V Ojeda, Noel C Perkins, Stephen M Cain

**Affiliations:** Department of Mechanical Engineering, University of Michigan, Ann Arbor, MI 48109, USA; lojeda@umich.edu (L.V.O.); ncp@umich.edu (N.C.P.); smcain@umich.edu (S.M.C.)

**Keywords:** wearable sensors, ZUPT, sensor requirements, gait

## Abstract

Researchers employ foot-mounted inertial measurement units (IMUs) to estimate the three-dimensional trajectory of the feet as well as a rich array of gait parameters. However, the accuracy of those estimates depends critically on the limitations of the accelerometers and angular velocity gyros embedded in the IMU design. In this study, we reveal the effects of accelerometer range, gyro range, and sampling frequency on gait parameters (e.g., distance traveled, stride length, and stride angle) estimated using the zero-velocity update (ZUPT) method. The novelty and contribution of this work are that it: (1) quantifies these effects at mean speeds commensurate with competitive distance running (up to 6.4 m/s); (2) identifies the root causes of inaccurate foot trajectory estimates obtained from the ZUPT method; and (3) offers important engineering recommendations for selecting accurate IMUs for studying human running. The results demonstrate that the accuracy of the estimated gait parameters generally degrades with increased mean running speed and with decreased accelerometer range, gyro range, and sampling frequency. In particular, the saturation of the accelerometer and/or gyro induced during running for some IMU designs may render those designs highly inaccurate for estimating gait parameters.

## 1. Introduction

Studies of running biomechanics suggest that measured kinematic parameters (e.g., joint angles, stride frequency, stride length) may lead to the insight necessary to improve running performance and reduce injury risk [1,2]. Miniature inertial measurement units (IMUs) are an attractive option for analyzing human performance outside of traditional laboratory environments due to their relatively low cost, simple setup, and portability [3]. In one application, foot-mounted IMUs provide three-dimensional foot accelerations and angular rotational velocities from which foot trajectories (and associated gait parameters) are derived during walking/running [4,5,6,7,8,9,10]. Doing so requires minimizing the accumulated drift error in the estimated foot velocity and position using the so-called “zero-velocity update” (ZUPT) method [4,5,11]. The ZUPT method exploits the fact that the foot is nearly stationary at some time during the stance phase and uses that condition to estimate the foot velocity drift error for each gait cycle.

Prior studies confirm that the ZUPT method yields accurate foot trajectory estimates for walking gait [9,12,13] and running gait with modest speeds (up to 4.36 m/s) [14]. However, little research addresses the requirements that ensure accurate trajectory estimates, particularly at faster running speeds, such as those observed in competitive middle- and long-distance running (up to 6.5 m/s) [15,16,17]. One limitation is that the sensor never achieves exactly zero-velocity, even in walking [18], and this assumption becomes increasingly suspect for faster running speeds. Another limitation lies with the range and sampling frequency of the inertial sensors themselves. Bailey and Harle [19] investigated the effect of IMU sampling frequency and accelerometer range on foot trajectory estimates and found that errors increase with increased running speed and decreased sampling frequency. While the errors observed in [19] were relatively small, the study considered modest running speeds (2.3–3.4 m/s) that are well below those of typical competitive middle- and long-distance running (up to 6.5 m/s). Additionally, the experiments in [19] were conducted on a treadmill rather than running overground which may also have influenced the conclusions. For example, significant differences may arise in gait kinematics when comparing walking on a treadmill versus overground [20]. Additionally, in a pilot study [21], the authors evaluated estimates of IMU-derived running speed using a treadmill. They observed that fluctuations in the treadmill belt speed, especially at higher running speeds with accompanying larger ground reaction forces, generated significant discrepancies between the reported belt speed and IMU-derived estimates of running speed. The speed fluctuations (i.e., accelerations) of the treadmill belt render it a non-inertial frame; thus, IMU-measured accelerations and angular velocities cannot be directly integrated to yield accurate estimates of foot velocity and position relative to the belt (as assumed using the ZUPT method). These treadmill-based limitations were a primary factor in modifying the pilot study protocol to the overground-based protocol of the study presented in this paper. Recently, Mitschke, et al. [22] examined the impact of accelerometer range on IMU-derived estimates of stride length, velocity, and tibial acceleration for overground running. They found no significant degradation in stride parameter estimates when the accelerometer range was ±32 g or greater, but significant degradation with smaller accelerometer ranges. Similar to [19], the study [22] considered only modest running speeds (up to 3.6 m/s) and did not disclose the fundamental reasons for the inaccurate estimates within the ZUPT method. In addition to the limitations imposed by accelerometer range and sampling frequency considered in these prior studies, we hypothesize that gyro range may also impose limitations on achieving accurate foot trajectory estimates for running. The effect of gyro range on stride estimates has likely not been studied previously because many commercial IMUs are unlikely to experience gyro saturation at the modest speeds observed in past studies. However, we hypothesize that the effects of gyro range on these estimates will become increasingly important at higher running speeds.

The objective of this study is to reveal the impact of an IMU’s accelerometer range, gyro range, and sampling frequency on estimated stride parameters (i.e., stride length, stride angle, and total distance traveled) during overground walking and running up to competitive distance running speeds (up to 6.4 m/s) and over a wide range of sensor ranges and sampling frequencies typically found in commercially-available IMUs. This study also addresses the impact of gait speed on the estimated distance traveled in the presence of no saturation of the IMU signals. The novelty and contribution of this work are that it: (1) quantifies these effects at mean speeds commensurate with competitive distance running (up to 6.4 m/s); (2) identifies the root causes of inaccurate foot trajectory estimates obtained from the ZUPT method; and (3) offers important engineering recommendations for selecting accurate IMUs for studying human running. The results of this study will aid coaches and researchers in selecting appropriate IMUs to study stride parameters in outdoor environments and at speeds up to 6.4 m/s using the ZUPT method.

## 2. Materials and Methods

### 2.1. Subjects and Experimental Protocol

**Figure 1:**
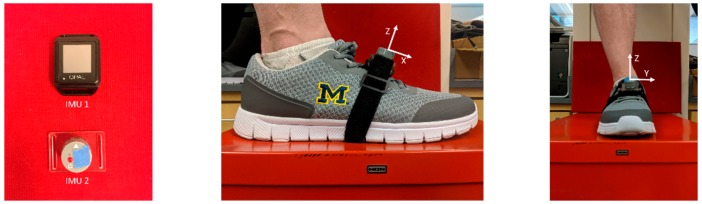
Two inertial measurement unit (IMU) designs and the means of attachment to a foot. (**a**) IMU 1 (Opal, APDM, left) and IMU 2 (custom design, Insight Sports, Ltd., right); (**b** and **c**) Side and front views of attachment of both IMUs to the instep via a Velcro strap. Sensor axes are denoted by X, Y, and Z (X and Z largely lie in foot sagittal plane and Y largely points to subject’s left). Note that these axes are illustrated to aid interpretation of the raw data signals presented in Figures 4 and 8, and that the zero-velocity update (ZUPT) method herein does not require specific sensor alignment to anatomical axes.

Six healthy subjects (3 female, 3 male; mean (standard deviation) age 24.2 (±6.0) years, height 1.71 (±0.12) m, mass 68.4 (±15.2) kg) were recruited for this study. All subjects verified that they felt capable of completing the experimental protocol. Informed consent was obtained from all subjects and the study was approved by the University of Michigan IRB. Two IMU designs were employed. The first (IMU 1) provides a high accelerometer range and the second (IMU 2) provides a high sampling frequency as reported by the IMU specifications in Table 1. Each subject wore both IMUs strapped together and placed on the bridge of both feet as shown in Figure 1 (only right foot shown) and secured with a strap and tape to limit their movement with respect to each other and to the foot.

We employed a method similar to [4] to assess the accuracy of ZUPT-based foot trajectory estimates outside of laboratory environments as follows. Each subject completed ten straight 100-meter trials on (level) asphalt. The subjects completed the first two trials at a perceived slow walk and fast walk, respectively. For the third through to the tenth trial, subjects ran at increasing speeds from a perceived slow jog (third trial) up to maximal sprint (tenth trial). For each trial, the subjects started at rest with the front of both shoes aligned with the start line. The subjects then walked/ran 100 meters and stopped with the front of both shoes aligned with the finish line. Rest between trials was self-selected by the subjects.

Since all trials started and ended with the subject at rest, the time to complete each trial was readily identified from the start and end of significant acceleration (magnitude). The known 100-meter distance traveled was divided by the trial time to yield the mean speed for each trial. We employed the mean speed as an independent variable in the analyses below.

### 2.2. Overview of the ZUPT Method

While the ZUPT method is generally known, we provide an overview for the reader’s benefit in following the discussions offered later in this paper. The ZUPT method used here draws largely from [4] and [9]. The method begins with estimating the instantaneous orientation of the IMU relative to an inertial frame as further detailed in [23]. The orientation is described by the rotation matrix, R, that defines the orientation of the sensor’s three orthogonal axes [${\hat{i}}_{s}$, ${\hat{j}}_{s}$,${\hat{k}}_{s}$] (corresponding to x, y, and z sensor axes, respectively) relative to the orthogonal axes [${\hat{i}}_{w}$, ${\hat{j}}_{w}$,${\hat{k}}_{w}$] of a world (i.e., inertial) frame (corresponding to x, y, and z world axes, respectively, with z pointing in the direction of gravity) per:

(1) $$\left[\begin{array}{c} {\hat{i}}_{w}\\ {\hat{j}}_{w}\\ {\hat{k}}_{w} \end{array}\right]=R\left[\begin{array}{c} {\hat{i}}_{s}\\ {\hat{j}}_{s}\\ {\hat{k}}_{s} \end{array}\right]$$

Following [23], the angular velocity is integrated to estimate R at each time step with a Kalman filter used to correct drift error in the tilt angle. The three components of IMU-measured acceleration ($a_{xs}$, $a_{ys}$, $a_{zs}$) yield the acceleration components in the world frame ($a_{xw}$, $a_{yw}$, $a_{zw}$) through:

(2) $$\left[\begin{array}{c} a_{xw}\\ a_{yw}\\ a_{zw} \end{array}\right]=R\left[\begin{array}{c} a_{xs}\\ a_{ys}\\ a_{zs} \end{array}\right]-\left[\begin{array}{c} 0\\ 0\\ g \end{array}\right]$$

where g is the acceleration of gravity. These acceleration components in the inertial frame are used to estimate velocity in the inertial frame using the fact that the foot-mounted IMU returns to zero-velocity during the stance phase as described below.

First, zero-velocity times are identified as times of minimum angular velocity magnitude during the stance phase. A stride is defined by the time interval between two successive zero-velocity times $t_{n-1}<t<t_{n}$. Thus, the stride time is:

(3) $$t_{s}=t_{n}-t_{n-1}$$

and the number of data samples, l, in each stride is:

(4) $$l=t_{s}\;\times \;F_{s}+1$$

where $F_{s}$ is the sampling frequency of the IMU. For each stride, the initial velocity is set to zero at $t_{n-1}$ and the acceleration components in the inertial frame (2) are integrated to estimate the velocity components in the inertial frame (${\overset{´}{v}}_{xw}$, ${\overset{´}{v}}_{yw}$, ${\overset{´}{v}}_{zw}$). Because it is assumed that the velocity at $t_{n}$ returns to zero, the estimated velocity error (per sample) in the stride is:

(5) $${\vec{V}}_{error}=\left[\begin{array}{c} {\overset{´}{v}}_{xw}\\ {\overset{´}{v}}_{yw}\\ {\overset{´}{v}}_{zw} \end{array}\right]\left(t=t_{n}\right)\times \frac{1}{l-1},$$

assuming linear drift. For each sample number, *k*$\;\in \left(1,l\right)$, within the stride (e.g., k = 3 for third sample in stride), a linear velocity drift error correction is applied to each of the three world frame velocity components:

(6) $$\left[\begin{array}{c} v_{xcorr}\\ v_{ycorr}\\ v_{zcorr} \end{array}\right]=\left[\begin{array}{c} {\overset{´}{v}}_{xw}\\ {\overset{´}{v}}_{yw}\\ {\overset{´}{v}}_{zw} \end{array}\right]-{\vec{V}}_{error}\times \left(k-1\right).$$

**Figure 2:**
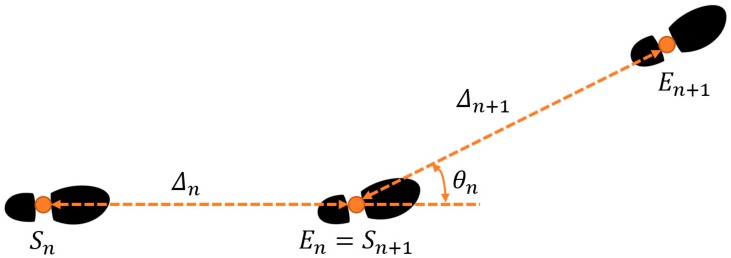
Illustration of stride length , Δ, and stride angle, θ. Orange dots on footprints represent consecutive zero-velocity times.

The corrected velocity for the whole trial is integrated to estimate the foot position throughout the trial. The position estimates are segmented by zero-velocity times and used to estimate the stride length and stride angle as follows. Figure 2 gives a two-dimensional illustration of the stride length and angle.

Identified zero-velocity times define the start position, ${\vec{S}}_{n}$, and end position, ${\vec{E}}_{n}$, of the $n^{th}$ stride as given by:

(7) $${\vec{S}}_{n}=\left[\begin{array}{c} S_{xw,n}\\ S_{yw,n}\\ S_{zw,n} \end{array}\right],$$

(8) $${\vec{E}}_{n}=\left[\begin{array}{c} E_{xw,n}\\ E_{yw,n}\\ E_{zw,n} \end{array}\right],$$

which denote the X, Y, and Z components of the start and end positions in the world frame for the $n^{th}$ stride. The stride length, Δ, is calculated as the total three-dimensional displacement of the IMU (and thus the foot) between the start and end of the stride. We note that in many biomechanical studies, stride length often refers to only the anterior–posterior component of the foot displacement; however, in using this technology for biomechanical analyses, there is a precedent to use alternative definitions of stride length, such as the total horizontal displacement of the foot, such as in [14]. Other studies make assumptions about the sensor/foot orientation, such as assuming a particular sensor axis is perfectly aligned with the medial–lateral axis of the subject [22] or impose a level ground assumption to constrain vertical drift [24]. While such assumptions may be useful in simplifying calculations or in interpreting some of the other foot parameters obtainable using similar ZUPT method applications (e.g., if interested in the foot roll and pitch angles, precise alignment of sensor and anatomical axes may be helpful or even necessary), we do not impose such restrictions in our method (e.g., our method can be used for non-level walking/running and our method does not require any alignment between the sensor and anatomical axes). In particular, the stride length (as defined for this study) for the $n^{th}$ stride is:

(9) $$\Delta_{n}=\sqrt{{\left(E_{xw,n}-S_{xw,n}\right)}^{2}+{\left(E_{yw,n}-S_{yw,n}\right)}^{2}+{\left(E_{zw,n}-S_{zw,n}\right)}^{2}}.$$

The stride angle, θ, is calculated as the three-dimensional angle between successive strides’ vectors. First, we define the stride vector for the $n^{th}$ stride as:

(10) $${\vec{D}}_{n}=\left[\begin{array}{c} E_{xw,n}-S_{xw,n}\\ E_{yw,n}-S_{yw,n}\\ E_{zw,n}-S_{zw,n} \end{array}\right].$$

The $n^{th}$ stride angle is thus computed as:

(11) $$\theta_{n}=tan^{-1}\left(\frac{\left|{\vec{D}}_{n}\times {\vec{D}}_{n+1}\right|}{{\vec{D}}_{n}\cdot {\vec{D}}_{n+1}}\right),$$

with × and · being the standard vector cross and dot products, respectively. We again note that the estimated stride parameters used in this study (stride length and stride angle) do not require any assumptions of a particular alignment of the sensor axes on the foot.

### 2.3. Data Processing and Analysis

Each IMU’s acceleration and angular velocity data were used to estimate the foot’s three-dimensional trajectory throughout each trial using the ZUPT method described above. The stride lengths were added (for both the left and right foot and then averaged) to yield the estimated total distance traveled (*D_calc_*) during each trial. The cumulative distance error:

*D_err_* = (*D_calc_*/*D_truth_* − 1) × 100%, (12)

is reported for each trial, where *D_truth_* is the known distance traveled (100 meters in this study).

To investigate the effect of accelerometer range, the raw accelerometer data from IMU 1, that possesses an acceleration range ±200 g, were numerically truncated to seven smaller ranges; namely 100, 75, 50, 24, 16, 10, and 6 g. For example, to investigate the effect of a 16 g accelerometer, any acceleration outside the range of −16 g < *a* < 16 g was set to the corresponding limit (−16 g or 16 g) to simulate sensor saturation at that limit. These seven ranges were chosen because they are typical of commercial IMU designs. Note that the acceleration data never exceeded ±100 g in any trial and thus we used the data with the ±100 g accelerometer range as the baseline for this analysis. After the raw accelerometer data were modified in this manner, the ZUPT method was used (with the modified accelerometer data and raw angular velocity data as input) to estimate the foot trajectories. The cumulative distance error (12) was computed as a function of the mean speeds for each accelerometer range. These data were then fit to a linear mixed-effects model [25] to test the statistical significance of the following: (1) the effect of mean gait speed on the cumulative distance error with no accelerometer saturation (i.e., using the 100 g range accelerometer), and (2) the effect of smaller accelerometer ranges on these estimates (i.e., using the 75, 50, 24, 16, 10, and 6 g range accelerometers). The statistical model and its full results are detailed in Appendix A. Similarly, to investigate the effect of gyro range, the raw angular velocity data from IMU 1, which possesses an angular velocity range of ±2000 deg/s, were also numerically truncated to four smaller ranges; namely 1500, 1000, 750, and 500 deg/s (i.e., ranges common in commercially available IMU designs). For this analysis, we used the data with the ±2000 deg/s gyro range as the baseline. After the raw angular velocity data were modified in this manner, the ZUPT method was used (with the modified angular velocity data and raw accelerometer data as input) to estimate the foot trajectories and cumulative distance error as described above. An analogous statistical model to the one described above was also employed, but investigating gyro range instead of accelerometer range.

Additionally, for both accelerometer and gyro range effects, the amount of data that was lost due to truncation was quantified. To accomplish this, we compared the integrated area under the truncated signal and that of the non-truncated signal. In particular, to quantify the data lost due to accelerometer saturation, we defined the percent data loss as:

(13) $$L_{data}=\left(1-\frac{\int \left|{\vec{a}}_{trun}\right|dt}{\int \left|{\vec{a}}_{non}\right|dt}\right)\times 100\%,$$

where $\left|{\vec{a}}_{trun}\right|$ is the acceleration magnitude of the truncated signal, $\left|{\vec{a}}_{non}\right|$ is the corresponding acceleration magnitude of the non-truncated signal, and dt is the time per sample. The integration is over the length of the trial. The percent data loss due to angular velocity saturation was defined analogously.

Note that IMU 1 was specifically chosen for studying the effects of accelerometer and gyro ranges and we verified that all data were within the design ranges (±200 g and ±2000 deg/s) for IMU 1 in all trials. By contrast, accelerometer saturation arose in IMU 2 (±32 g) at higher running speeds. However, IMU 2 was specifically chosen to study the effect of sampling frequency due to the sampling frequency limitations of IMU 1 (128 Hz). IMU 2 possesses a sampling frequency of 1000 Hz, far beyond the minimum 250 Hz rate recommended in [19] for obtaining accurate foot position and velocity estimates using the ZUPT method in running at speeds up to 3.4 m/s. For the purpose of studying the effect of sampling frequency, the accelerometer and gyro data from IMU 2 (1000 Hz) were also down-sampled to four smaller sampling frequencies (500, 250, 125, and 62.5 Hz) typical of commercial IMU designs. To that end, we employed two down-sampling methods as further described in the Results section. For these analyses, we used the data with the 1000 Hz sampling frequency as the baseline.

In addition to studying the cumulative distance error, we also report stride-to-stride variations in the differences of individual stride length and stride angle estimates as defined above (e.g., the standard deviation of the stride length difference over a trial) as these data also reveal important conclusions. To this end, the stride length difference for a particular stride and trial (e.g., stride 2 for subject 1 and trial 1) was defined as:

(14) $$\Delta_{dif}=\Delta_{base}-\Delta_{est},$$

where the $\Delta_{base}$ is the estimated stride length using the baseline (non-saturated/non-downsampled) IMU data and $\Delta_{est}$ is the estimated stride length using the truncated or downsampled data generated as discussed above.

## 3. Results

### 3.1. Effect of Accelerometer Range

**Figure 3:**
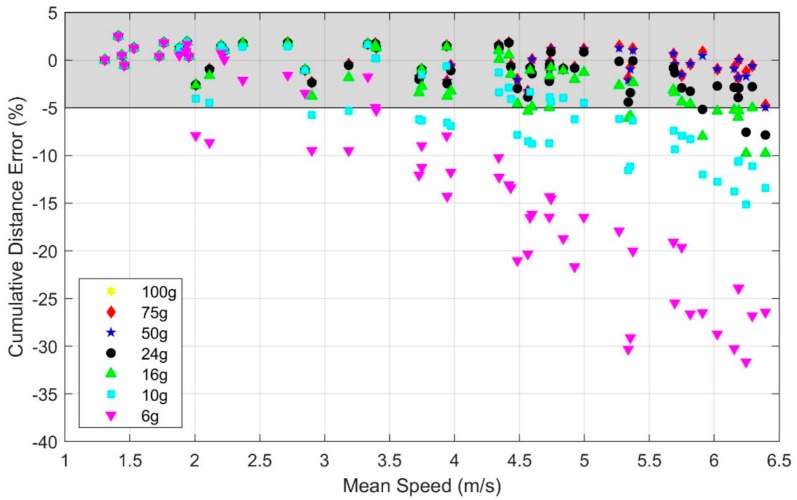
Effect of accelerometer range and mean running speed on the cumulative distance error. Note that errors arising from the 100, 75, and 50 g accelerometers are indistinguishable on this scale. The shaded region indicates estimates within ±5% error.

The statistical analysis (Table A1 of Appendix A) reveals a significant effect of speed on cumulative distance error (*p* < 0.01) with the 100 g accelerometer range despite no observed accelerometer or gyro signal saturation in any of the trials with this range. Additionally, the statistical analysis reveals that using an accelerometer range of 24 g or below leads to significantly greater degradation of the estimated distance traveled with speed (*p* < 0.01 for 24 g, *p* < 0.001 for 16 g, 10 g, and 6 g) relative to the 100 g range. The accelerometer ranges of 75 g and 50 g revealed no statistically significant effects compared to 100 g. These findings are observable in Figure 3 which illustrates the cumulative distance error versus mean running speed for the original (100 g) accelerometer range and for each of the six truncated accelerometer ranges utilizing data from IMU 1. As illustrated, subjects achieved mean speeds up to 6.4 m/s with the upper end of this range, similar to speeds observed in elite distance running [15]. Because the peak accelerations rarely exceeded 50 g, the cumulative distance errors for the 100, 75, and 50 g accelerometer ranges are visually indistinguishable on this scale as expected from the statistical results. Additionally, note that the cumulative distance error results converge across accelerometer ranges with decreased running speeds because these speeds generally yield lower accelerations (e.g., for the slowest trial, accelerations never exceeded 6 g, yielding identical cumulative distance errors for all accelerometer ranges considered).

For walking and low running speeds (i.e., <2.2 m/s), the illustrated results largely confirm the cumulative distance errors reported by others for walking [4,9]. Additionally, for these low speeds, the IMU-estimated cumulative stride distances are nearly independent of accelerometer range. This is expected since the peak accelerations rarely exceeded 6 g for walking and low running speeds. However, for high running speeds, significant portions of each stride cycle generated accelerations larger than 6 g, leading to the large observable degradations in the estimated cumulative distance at the higher running speeds with decreased accelerometer range. Importantly, Figure 3 shows that accelerometers with ranges exceeding 50 g yield cumulative distance errors no greater than 5% for all mean speeds observed in this study (up to 6.4 m/s). Thus, depending on the accuracy needs for a particular use of these estimates, the ZUPT method may yield acceptable results (i.e., errors of 5% or less) even at the highest speeds observed in this study, provided no saturation arises in the accelerometer (and gyro) signals. By contrast, at the opposite extreme, errors exceeding 30% are observable for the 6 g accelerometer where significant saturation occurs.

**Figure 4:**
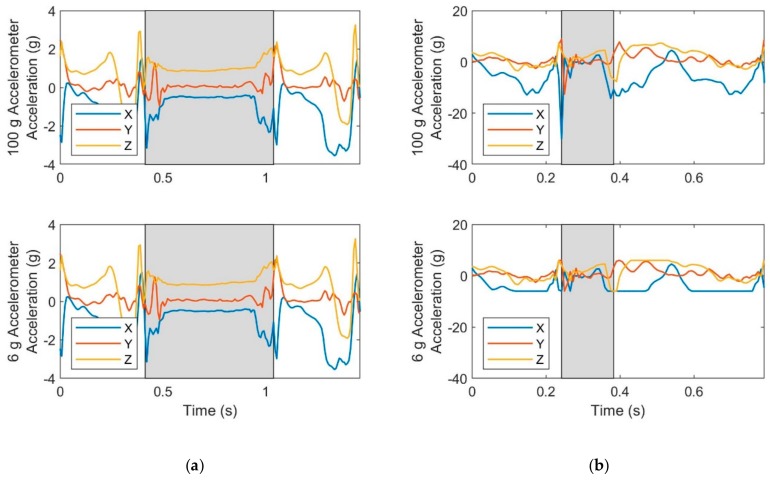
Effect of accelerometer range limits on acceleration data for a sample (**a**) walking trial (mean speed 1.4 m/s) and a sample (**b**) running trial (mean speed 5.8 m/s). Acceleration data is saturated when the slope is zero as is most apparent for the *X*-axis acceleration of the 6 g accelerometer for the running trial. Shaded areas indicate stance phase.

The degradation of estimates of the cumulative distance traveled with increased speed and decreased accelerometer range traces to saturation in the accelerometer signals. Figure 4 illustrates the effect of truncating the accelerometer range for sample walking (Figure 4a) and running (Figure 4b) trials. In the sample walking trial, the three acceleration components never exceed 6 g and therefore distance estimates based on any of the accelerometer ranges considered (6 g through 100 g) yield essentially identical results. However, in the sample running trial, the acceleration components often exceed 6 g and for significant portions of the gait cycle. The data loss leads to significant foot trajectory errors largely due to how the ZUPT method corrects for velocity drift error as described in detail in the Discussion.

**Figure 5:**
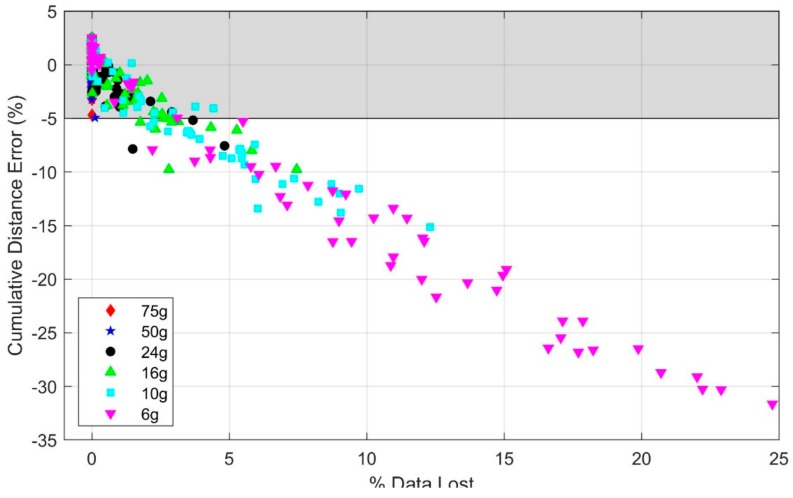
Cumulative distance error versus percentage of acceleration data lost due to saturation for all trials and for each accelerometer range. The shaded region indicates estimates within ±5% error.

We note that the presence of saturation does not necessarily lead to poor estimates. In particular, acceptable results may still be obtainable if the amount of data that is lost due to saturation remains small. To illustrate what might be “acceptable” levels of saturation for the accelerometer range, we further quantified the amount of data that is lost due to saturation for each trial and accelerometer range and present the relationship of the cumulative distance error and amount of data lost due to saturation in Figure 5. These results show that the cumulative distance errors remain below 5% when the percentage of acceleration data lost due to saturation is below 1.5%, irrespective of the accelerometer range.

**Figure 6:**
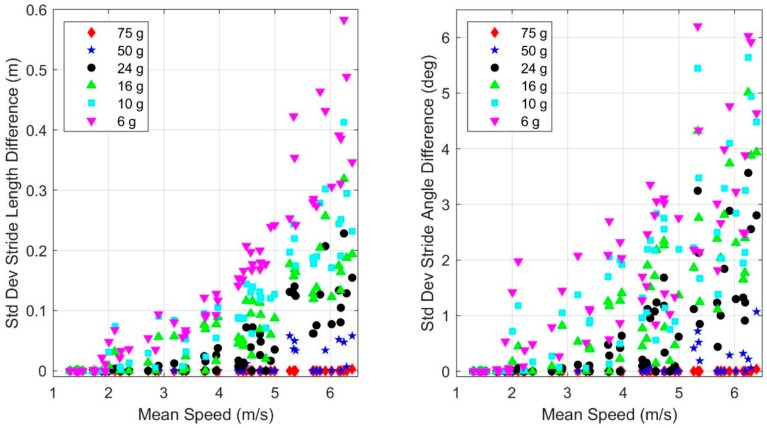
Dependence of the standard deviation of difference in (**a**) stride length and (**b**) stride angle estimates with mean speed and accelerometer range. The differences are computed with respect to the results of the 100 g accelerometer as the benchmark.

Beyond studying errors in the cumulative distance traveled, we also considered differences in stride length and stride angle estimates on a stride by stride basis. To this end, we compared the estimated length and angle of each stride (where stride angle refers to the angle between successive strides as defined in the Methods) using truncated accelerometer data to the same quantities estimated from untruncated accelerometer data, employing the 100 g accelerometer as the benchmark. Figure 6 illustrates the standard deviation of the resulting differences in the individual stride length (Figure 6a) and stride angle (Figure 6b) estimates. When the variation is large, the agreement between truncated and baseline estimates of the given parameter for individual strides is small. These variations increase strongly with mean speed and decreased accelerometer range. As both mean speed and accelerometer range contribute to accelerometer saturation, they also significantly impact the estimates of these metrics on an individual stride basis.

### 3.2. Effect of Gyro Range

**Figure 7:**
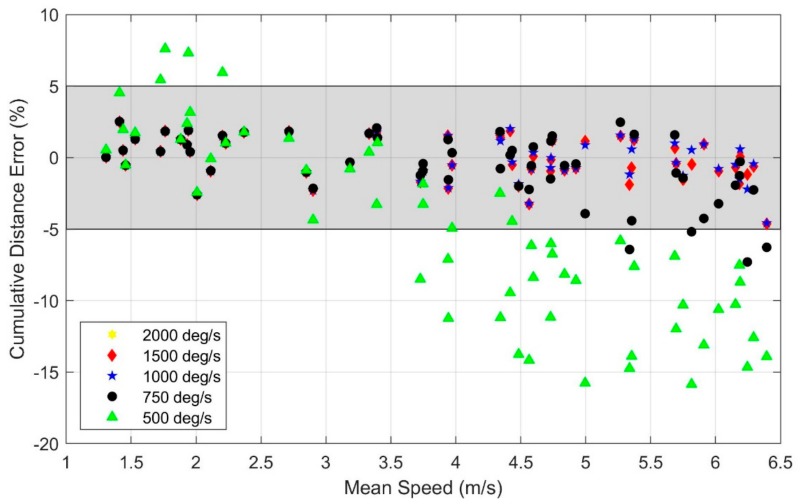
Effect of gyro range and mean running speed on the cumulative distance error. Note that distance errors arising from the 2000 and 1500 deg/s gyros are indistinguishable on this scale. The shaded region indicates estimates within ±5% error.

The statistical analysis (Table A2 of Appendix A) reveals a significant effect of speed on cumulative distance error (*p* < 0.01) with the 2000 deg/s gyroscope range despite no observed accelerometer or gyro signal saturation in any of the trials with this range. Additionally, the statistical analysis reveals that using a gyro range of 750 deg/s or below leads to significantly greater degradation of the estimated distance traveled with speed (*p* < 0.05 for 750 deg/s, *p* < 0.001 for 500 deg/s) versus the 2000 deg/s range. Gyro ranges of 1500 deg/s and 1000 deg/s reveal no statistically significant effects compared to 2000 deg/s. These findings are observable in Figure 7 which illustrates the cumulative distance error versus mean running speed for the five gyro ranges considered utilizing data from IMU 1. Because the angular velocities rarely exceeded 1000 deg/s, the distance error is nearly the same for the 2000, 1500, and 1000 deg/s range gyros. The distance errors remain within 5% for all gyro ranges of at least 1000 deg/s for the entire range of mean speeds studied herein (up to 6.4 m/s).

**Figure 8:**
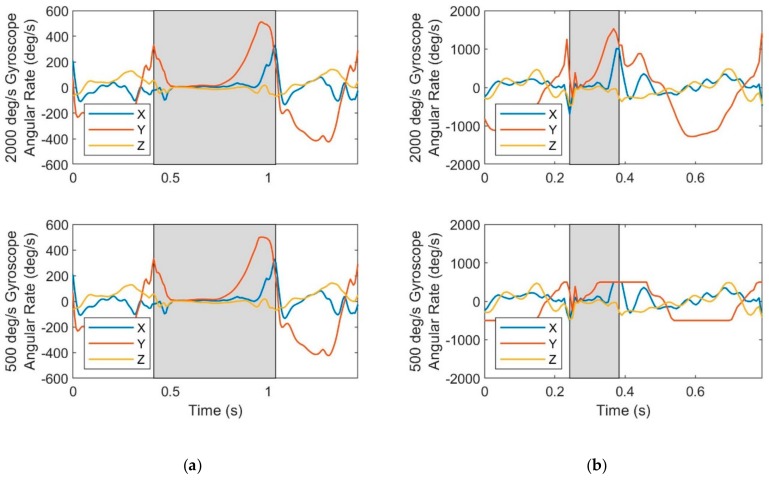
Effect of gyro range limits on angular velocity for a sample (**a**) walking trial (mean speed 1.4 m/s) and a sample (**b**) running trial (mean speed 5.8 m/s). Angular velocity data is saturated when the slope is zero as is most apparent for the *Y*-axis angular velocity of the 500 deg/s gyro for the running trial. Shaded areas indicate stance phases.

As in Figure 3, the cumulative distance traveled is underestimated at faster (running) speeds and this underestimation increases with speed and decreased gyro range. However, the cumulative distance traveled is often overestimated at slower (walking) speeds and this overestimation increases with decreased gyro range; observe the slower (walking) trials with a 500 deg/s range gyro. The underestimation versus overestimation traces to saturation in distinct portions of the stride cycle as revealed in Figure 8 for sample walking (Figure 8a) and running (Figure 8b) trials. Observe in Figure 8a that the *Y*-axis angular velocity for the 500 deg/s gyro exhibits saturation during a modest fraction of the stance phase near toe-off (end of the stance phase) for walking. By contrast, Figure 8b reveals that for maximal sprinting, the same angular velocity component saturates during toe-off, heel-strike (beginning of the stance phase), and for a significant portion of the swing phase. The portion of the stride cycle in which data is lost leads to overestimation versus underestimation because of how it impacts the ZUPT algorithm as described in detail in the Discussion.

**Figure 9:**
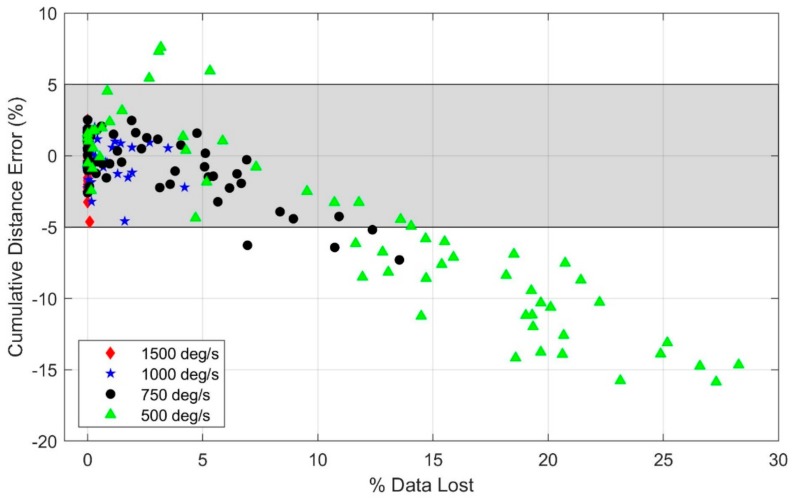
Cumulative distance error versus percentage of angular velocity data lost due to saturation for all trials and for each gyro range. The shaded region indicates estimates within ± 5% error.

As with the accelerometer, we note that the presence of saturation in the gyro does not necessarily lead to poor estimates; in particular, acceptable results may still be obtainable if the amount of data that is lost due to saturation remains small. To illustrate what might be “acceptable” levels of saturation for gyro signals, we further quantify the amount of data that is lost due to saturation for each trial and gyro range and present the relationship of cumulative distance error and amount of data lost due to saturation in Figure 9. These results show that cumulative distance errors remain below 5% when the percentage of angular velocity data lost due to saturation is below 2.6%, regardless of the gyro range.

**Figure 10:**
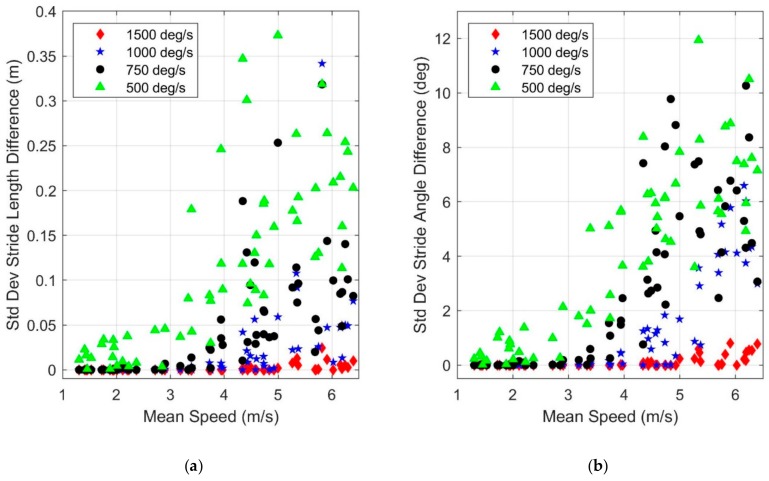
Dependence of the standard deviation of the difference in (**a**) stride length and (**b**) stride angle estimates with mean speed and gyro range. The differences are computed with respect to the results of the 2000 deg/s gyro as the benchmark.

As in the previous section, we also considered differences in stride length and stride angle estimates on a stride by stride basis. To this end, we compared the estimated length and angle of each stride (where stride angle refers to the angle between successive strides as defined in the Methods) using truncated gyro data to the same quantities estimated from untruncated gyro data, employing the 2000 deg/s gyro as the benchmark. Figure 10 illustrates the standard deviation of the resulting differences in the individual stride length (Figure 10a) and stride angle (Figure 10b) estimates. When the variation is large, the agreement between the truncated and baseline estimates of the given parameter for individual strides is small. These variations increase strongly with mean speed and decreased gyro range. As both mean speed and gyro range contribute to gyro saturation, they also significantly impact the estimates of these metrics on an individual stride basis.

### 3.3. Effect of Sampling Frequency

Sampling methods can vary widely in commercial IMUs. In particular, one or more filters are commonly employed within the IMU hardware and/or software before data is output at the IMUs specified sampling frequency. Therefore, an IMU having a higher sampling frequency (specification) does not necessarily imply it will lead to superior estimates of stride parameters in the context of this study. Because the filters and sampling methods are generally hidden to the user and vary between manufacturers, we considered the effect of sampling frequency by studying two simple sampling methods, including both an extreme method (no filtering before down-sampling) and a common method (low pass filter before down-sampling). For both methods, data from IMU 2 was utilized as that IMU design yields data at a high (1000 Hz) sampling frequency. Neither sampling method demonstrated a statistically significant effect for the interaction of speed and sampling frequency on the cumulative distance error except for the most extreme downsampling used in this study (Method 1 at the lowest sampling frequency). See Appendix A for full statistical results. Because this one exception represents a most unrealistic scenario and because no other sampling method and sampling frequency combinations studied herein revealed statistically significant effects for this interaction, we offer no further results for this effect. However, significant differences do arise in the estimated stride lengths and stride angles of the individual strides as reported below.

**Figure 11:**
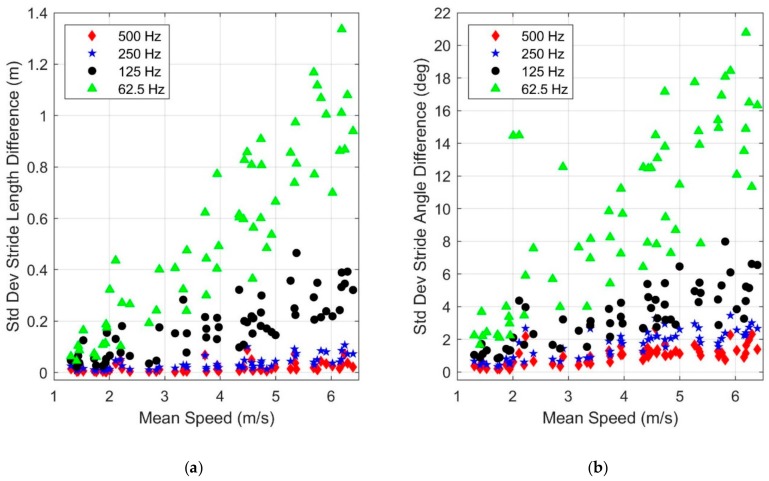
Dependence of the standard deviation of the difference in (**a**) stride length and (**b**) stride angle estimates with mean speed and sampling frequency. Method 1: down-sampling without filtering. The differences are computed with respect to the results of the 1000 Hz sampling frequency as the benchmark.

Method 1 constitutes simple down-sampling performed without filtering (e.g., when down-sampling from 1000 to 500 Hz, every other sample is retained). This overly simplistic approach introduces aliasing effects and hence sub-optimal results [26,27]. Figure 11 illustrates the standard deviation of differences in stride length (Figure 11a) and stride angle (Figure 11b) estimates using Method 1 as functions of both mean speed and sampling frequency. The differences are with respect to the same quantities computed using the original data (i.e., data sampled at 1000 Hz). The standard deviation of the difference from simple down-sampling quickly grows (i.e., increasing variation) with increasing mean speed and decreasing sampling frequency. For example, at the lowest sampling frequency (62.5 Hz), the standard deviation in stride length difference becomes a significant fraction of the stride length at higher mean speeds. Thus, the reliability of these measures is significantly impacted by both mean speed and sampling frequency.

**Figure 12:**
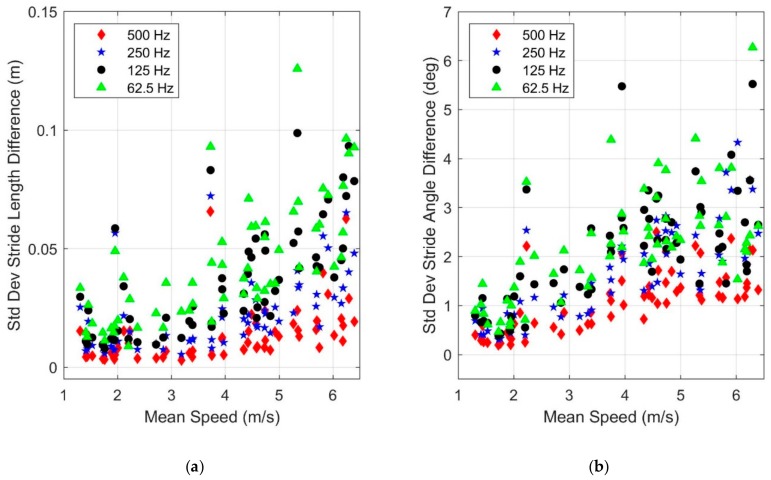
Dependence of the standard deviation of the difference in (**a**) stride length and (**b**) stride angle estimates with mean speed and sampling frequency. Method 2: low pass filtering before down-sampling (using MATLAB^TM^ decimate function). The differences are computed with respect to the results of the 1000 Hz sampling frequency as the benchmark. Note the differences in the *y*-axis scales compared to Figure 11.

Method 2 follows a more common strategy known as decimation [28,29], which consists of low pass filtering prior to down-sampling. We used the decimate function in MATLAB^TM^ [30] which utilizes a low pass Chebyshev Type I filter (infinite impulse response, order 8) before down-sampling the data. Figure 12 illustrates the results from Method 2, analogous to those of Method 1. The results still illustrate increased differences in estimates with increased speed and decreased sampling frequency, but significantly less than that observed using Method 1 (Figure 11). For example, the variation of the stride length difference is reduced by nearly a factor of five (compare scales of Figure 11a and Figure 12a). These results suggest that sensor hardware that employs well-designed filters can significantly mitigate the adverse impact of limited sampling frequencies.

## 4. Discussion

Overall, this study highlights the importance of proper sensor selection in order to estimate accurate gait parameters from foot-mounted IMUs using the ZUPT method. Accurate estimates of the cumulative distance traveled are possible upon limiting acceleration and angular velocity saturation. Importantly, we observed that the cumulative distance error using the ZUPT method remains below 5% when acceleration saturation is limited to 1.5% and when angular velocity saturation is limited to 2.6%; refer to Figure 5 and Figure 9.

We also observed that gait parameter estimates degrade with higher mean speeds even without sensor saturation (*p* < 0.01); refer to Appendix A. However, the results confirm that ZUPT-based algorithms yield accurate estimates for some applications (i.e., less than 5% cumulative distance error) over the entire range of mean speeds studied herein (up to 6.4 m/s) contingent on the IMU design. Importantly, lower range inertial sensors yield significant errors in gait parameter estimates at higher mean speeds due to (increasingly larger) data saturation. Interestingly, saturation may produce both overestimates and underestimates of the cumulative distance traveled depending on which signal (acceleration or angular velocity) is saturated, in which part of the stride cycle most of the saturation occurs, and the mean speed. These errors arise from error sources within the ZUPT method (detailed in the Methods) as follows. The gyro data is employed to estimate the orientation of the IMU (via integrating angular velocity) and this is critical to accurately resolving the acceleration into the world frame. The orientation estimates are corrected for drift error using a Kalman filter based on the core assumption of zero-mean Gaussian gyro noise. However, this assumption is violated when the angular velocity saturates, leading to inaccurate estimates of orientation and thus improper resolution of acceleration in the world frame. Subsequent integration of poorly resolved (and even possibly saturated) acceleration yields inaccurate estimates of velocity and position. Additionally, even with proper IMU orientation estimates, saturation of accelerometer signals will create velocity drift errors that do not increase linearly in time between the zero-velocity update times as assumed in the ZUPT method.

These error sources suggest an intuitive explanation for why the estimated total distance traveled is increasingly underestimated with increased speed and decreased accelerometer range. In this study, the majority of acceleration data lost due to saturation was acceleration directed opposite to the direction of travel (i.e., deceleration). Consequently, the uncorrected velocity in the direction of travel was overestimated at the end of a stride. However, when the (linear) velocity-drift correction was then applied, it consistently lead to underestimated velocity in the direction of travel and corresponding underestimated stride length. By contrast, there is no parallel explanation for why saturated gyro data may lead to both over and under estimates of the stride length. In particular, note that saturation of gyro data creates errors farther upstream in the ZUPT algorithm and specifically in the orientation estimation. Errors in the orientation estimation may yield both over and under estimates of the stride length. Despite these several error sources, accurate velocity and position estimates are still obtained if either the percentage of the missing sensor data remains small (as described above) or if the saturation occurs along a sensor axis that does not contribute significantly to the estimate.

A naïve user may be tempted to conclude that it is always best to select an IMU with the largest ranges for acceleration and angular velocity to always avoid saturation. However, increased range often comes with the tandem penalty of reduced resolution (e.g., if range is increased, but bit resolution is not) as well as increased sensor noise, which may both defeat the apparent advantage of higher range sensors. Consequently, there could well be instances where an IMU possessing an accelerometer that admits minor saturation yields superior stride parameter estimates relative to one possessing a higher range accelerometer that admits no saturation. Of course, a superior concept is to employ multiple accelerometers and/or rate gyros with increasing (and even slightly overlapping) ranges, a concept not studied herein.

We note that in estimating the total distance traveled, symmetrically distributed stride length errors (i.e., some overestimated and some underestimated) may cancel, leading to accurate estimates of total distance traveled. Therefore, it is important to understand how individual stride length and angle estimates are affected by sensor parameters. We chose to study these effects using the reported standard deviation of the stride length (and stride angle) differences, where these differences were compared to the baseline estimates (using the maximal sensor parameters). By using both the cumulative error in the distance traveled and the standard deviations of stride length and stride angle differences, we were able to reach sound conclusions of how IMU parameters affect individual stride length estimates as reported herein despite not explicitly having stride by stride ground truth data. In particular, we note that standard deviations of stride length and stride angle differences appear to converge as sensor parameters (ranges and sampling frequency) approach the nominal parameters for that sensor (Figure 6, Figure 10, Figure 11 and Figure 12). This apparent convergence suggests that (as one would expect) the baseline estimates are likely the best available estimates and thus the differences likely correspond to degradations in the estimates. However, we also acknowledge that independent ground truth estimates of individual stride lengths and angles are required to confirm this conclusion and that data was not available in the present study. The convergence in standard deviations also suggests that improvements in estimates of individual stride parameters when going beyond the ranges and sampling frequencies utilized by the sensor in this study will be minor compared to the degradation effects due to sensor limitations demonstrated in this study.

For these experiments, sampling frequency showed no significant impact on estimates of the total distance traveled (except in one limited condition); however, it significantly impacted estimates of the individual stride parameters (stride length and stride angle). In particular, the variance of the individual stride parameters was significantly influenced by the filtering/sampling method employed. This effect was demonstrated using two simple down-sampling methods. While this analysis demonstrated differences in stride parameter estimates with a reduction of the sampling frequency on a single IMU, caution must be exercised when comparing sampling frequencies between IMU designs. Many factors of IMU design in addition to sampling frequency (e.g., sensor hardware, sensor placement) impact stride parameter estimates. Thus, it remains possible for an IMU with a modest sampling frequency (e.g., 128 Hz) to yield superior stride parameter estimates to another IMU design with a higher sampling frequency (e.g., 1000 Hz).

Finally, we describe several limitations of this study which we also believe do not alter the core conclusions. First, we acknowledge that the sensors available for this study both had limitations (in range and sampling frequency) that may affect the accuracy of the calculated stride metrics presented herein. Despite these limitations, we demonstrated important conclusions about the effects of the sensor parameters on the selected stride parameters. We duly note additional factors not studied herein that may impact the accuracy of stride estimates (e.g., sensor noise, sensor bandwidth, sensor resolution, Kalman filter tuning, etc.) that motivate future studies. Second, these experiments were conducted over a relatively short (100 m) distance. However, the results for the cumulative distance error are expected to hold for any (i.e., longer) distance because the cumulative distance error is equivalent to a percent error in the mean stride length estimates. Third, in the statistical analyses detailed in Appendix A, we did not evaluate subject-specific effects (i.e., we removed the effect of subjects on our results by treating subject as a random effect). Future studies could investigate the effect of subject demographics (e.g., weight, height, etc.) on estimates of gait parameters using the ZUPT method. Fourth, we note that our study is not well suited for traditional statistical power analyses. Thus, we remind the reader that caution should be employed when interpreting the effects that were not found to be significant in the statistical analyses as they may be subject to type-II statistical errors (i.e., an effect not found to be significant does not guarantee that there is no effect). However, we also note that the potential presence of type-II errors (for the effects not observed to be significant) in no way diminishes the importance of the effects that were observed to be significant and their associated conclusions. Fifth, we suggest that future studies investigate the impact of sensor properties on other stride parameters obtainable from the ZUPT method (e.g., foot clearance, foot roll angle, etc.).

## 5. Conclusions

Appropriate selection of the ranges and sampling frequencies of the inertial sensors embedded in IMUs is crucial for accurately estimating foot trajectories (hence gait parameters) from foot-mounted IMUs, and particularly for the speeds associated with competitive distance running. In this study, we investigated the effects of mean gait speed and sensor parameters on estimates of stride parameters. The novelty and contribution of this work are that it: (1) quantifies these effects at mean speeds commensurate with competitive distance running (up to 6.4 m/s); (2) identifies the root causes of inaccurate foot trajectory estimates obtained from the ZUPT method; and (3) offers important engineering recommendations for selecting accurate IMUs for studying human running. Estimates of the cumulative distance traveled (from the individual stride length estimates) degrade with speed; however, across the range of mean speeds studied here, estimates remained within 5% of ground truth if there was no or minor saturation of the accelerometer (1.5% or less) or gyro (2.6% or less) signals as defined herein. In particular, the reported experiments required accelerometer ranges of at least 50 g and gyro ranges of at least 1000 deg/s to avoid significant errors in estimates of the cumulative distance traveled for mean running speeds up to 6.4 m/s. Errors that arise due to sensor saturation trace to core assumptions that are violated in the underlying estimation procedure based on the ZUPT method (i.e., zero-mean Gaussian noise, zero-velocity, and linear velocity drift assumptions). For applications similar to the ones described in this paper, accurate results remain possible even with modest sampling frequencies (e.g., 128 Hz), provided well-designed filters are employed.

## Figures and Tables

**Table 1:** Specifications for IMU 1 and IMU 2.

	IMU 1	IMU 2
Model	Opal	Custom design
Manufacturer	APDM	Insight Sports, Ltd.
Sampling Frequency (Hz)	128	1000
Accelerometer Range (g)	±200	±32
Angular Gyro Range (deg/s)	±2000	±4000

## Appendix A—Full Statistical Results from Linear Mixed-Effects Models

We report results for four statistical analyses that reveal the effects of accelerometer range, gyro range, and downsampling by two methods. For each of the four analyses, we utilized a linear mixed-effects model to test the effects of speed, sensor parameter (sensor range or sampling frequency), and the combination of speed and sensor parameter on the cumulative distance error. In each model, we removed subject effects by treating the subject as a random effect. Because we are interested in the effect of mean speed on the cumulative distance error without saturation, we treated mean speed as a continuous variable and fixed effect. We are also interested in the interaction of mean speed and the sensor parameter (i.e., how speed affects the cumulative distance error for a change in the sensor parameter versus that effect for the baseline sensor parameter) and included this interaction as a fixed effect. The model also calculated the effects of the sensor parameter alone (effect of only sensor parameter independent of speed) and a *y*-axis intercept (cumulative distance error with a mean speed of 0 m/s) for the baseline sensor condition; however, we did not include those additional findings as they did not add to the conclusions or the interpretation of the presented results. In each analysis, we used estimates obtained from the original (non-truncated or non-downsampled) IMU data as the baseline. All statistical analyses were run in R statistical software using lme4 and lmerTest packages [25,31,32]. The function *lmer* was used to fit the models and the function *confint* was used to compute 95% confidence intervals for all estimates.

The four tables below present results from this model for each of the four analyses. The first (shaded) row in each table reports the effect of speed only on the cumulative distance error for the baseline sensor condition. This includes an estimated linear slope for the cumulative distance error versus speed (i.e., an estimated slope of 1%/(m/s) would indicate that the cumulative distance error increases by an estimated 1% for each 1 m/s increase in mean speed) for the baseline sensor condition where a negative value means that the error is negative as defined in this paper (i.e., underprediction of cumulative distance). This slope also represents the sensitivity of the cumulative distance error to mean speed. In the remaining (unshaded) rows, we report the interactions of mean speed and the sensor parameter (i.e., how a change to sensor parameters compared to the baseline sensor condition impacts the sensitivity of the cumulative distance error to mean speed). The estimated slopes in these rows denote the estimated *difference* in the slope of the cumulative distance error versus speed over that for the baseline condition. Thus, these entries report the additional error sensitivity to speed for the specified change in the sensor parameter relative to the baseline. For example, in Table A1, the estimated cumulative distance speed error is negative and it grows by −0.39%/(m/s) for the baseline sensor. The error sensitivity then grows (i.e., worsens) by an additional −0.57%/(m/s) for a sensor employing an accelerometer with a 24 g range. The third and fourth columns report the significance (*p*-value) and the 95% confidence interval for the estimated slope/error sensitivity.

**Table A1:** Effect of speed on the cumulative distance versus accelerometer range. First (shaded) row reports the estimated slope of the cumulative distance versus mean speed using the baseline sensor (100 g range accelerometer). Remaining (unshaded) rows report estimated differences in this slope for IMUs with indicated acceleration ranges compared to the baseline IMU (i.e., the increased error sensitivity to speed with the indicated reductions in acceleration range relative to the baseline range).

Speed Effect	Estimated Slope (%/(m/s))	*p*-Value	95% Conf. Int.
Baseline (100g)	−0.39	<0.01	(−0.65, −0.13)
75 g vs. Baseline	0.00	0.99	(−0.37, 0.37)
50 g vs. Baseline	−0.03	0.88	(−0.39, 0.34)
24 g vs. Baseline	−0.57	<0.01	(−0.94, −0.21)
16 g vs. Baseline	−1.08	<0.001	(−1.45, −0.72)
10 g vs. Baseline	−2.24	<0.001	(−2.60, −1.87)
6 g vs. Baseline	−5.70	<0.001	(−6.06, −5.33)

**Table A2:** Effect of speed on the cumulative distance versus gyro range. First (shaded) row reports the estimated slope of cumulative distance versus mean speed using the baseline sensor (2000 deg/s gyro). Remaining (unshaded) rows report estimated differences in this slope for IMUs with indicated angular velocity ranges compared to the baseline IMU (i.e., the increased error sensitivity to speed following the indicated reductions in angular velocity range relative to the baseline range).

Speed Effect	Estimated Slope (%/(m/s))	*p*-Value	95% Conf. Int.
Baseline (2000 deg/s)	−0.41	<0.01	(−0.68, −0.13)
1500 deg/s vs. Baseline	0.00	0.99	(−0.39, 0.39)
1000 deg/s vs. Baseline	0.05	0.81	(−0.34, 0.44)
750 deg/s vs. Baseline	−0.40	<0.05	(−0.80, −0.01)
500 deg/s vs. Baseline	−3.08	<0.001	(−3.47, −2.69)

**Table A3:** Effect of speed on the cumulative distance versus sampling frequency for Method 1 (downsampling). First (shaded) row reports the estimated slope of the cumulative distance traveled versus mean speed using the baseline sensor (1000 Hz). Remaining (unshaded) rows report the estimated differences in this slope for IMUs with the indicated sampling frequency compared to the baseline IMU (i.e., the increased error sensitivity to speed following the indicated reductions in sampling frequency relative to the baseline sampling frequency).

Speed Effect	Estimated Slope (%/(m/s))	*p*-Value	95% Conf. Int.
Baseline (1000 Hz)	−1.12	<0.001	(−1.48, −0.77)
500 Hz vs. Baseline	−0.09	0.73	(−0.59, 0.41)
250 Hz vs. Baseline	−0.13	0.63	(−0.63, 0.38)
125 Hz vs. Baseline	−0.26	0.32	(−0.76, 0.24)
62.5 Hz vs. Baseline	0.94	<0.001	(0.44, 1.44)

**Table A4:** Effect of speed on the cumulative distance versus sampling frequency for Method 2 (decimation). First (shaded) row reports the estimated slope of the cumulative distance traveled versus mean speed using the baseline sensor (1000 Hz). Remaining (unshaded) rows report the estimated differences in this slope for IMUs with the indicated sampling frequency compared to the baseline IMU (i.e., the increased error sensitivity to speed following the indicated reductions in sampling frequency relative to the baseline sampling frequency).

Speed Effect	Estimated Slope (%/(m/s))	*p*-Value	95% Conf. Int.
Baseline (1000 Hz)	−1.17	<0.001	(−1.50, −0.85)
500 Hz vs. Baseline	−0.01	0.96	(−0.47, 0.45)
250 Hz vs. Baseline	−0.03	0.90	(−0.49, 0.43)
125 Hz vs. Baseline	0.08	0.74	(−0.38, 0.54)
62.5 Hz vs. Baseline	0.12	0.60	(−0.33, 0.58)
